# Authors’ reply to Grundtvig Gram et al.

**DOI:** 10.1007/s10654-021-00775-z

**Published:** 2021-07-18

**Authors:** Bjørn Hofmann, Lynette Reid, Wendy A Rogers, Stacy M Carter

**Affiliations:** 1 Department in Health Sciences, Faculty of Medicine and Health Sciences, The Norwegian University of Science and Technology, Gjøvik, Norway; 2 Centre of Medical Ethics, Faculty of Medicine, The University of Oslo, Blindern, PO Box 1130, N-0318 Oslo, Norway; 3 Department of Bioethics, Faculty of Medicine, Dalhousie University, Halifax, Canada; 4 Department of Philosophy and Department of Clinical Medicine, Macquarie University, Sydney, NSW Australia; 5 Australian Centre for Health Engagement, Evidence and Values, School of Health and Society, University of Wollongong, Wollongong, NSW 2522 Australia

We thank Gram, Brodersen, and colleagues [1] for their engagement with our paper [2] on relevant perspectives in defining overdiagnosis. In this commentary we clarify the aims of that paper and respond to points they raise.

In our paper we do not propose a definition of overdiagnosis; rather we present an agreement among researchers, who have each proposed their own definition, about whose perspectives are relevant in addressing existing disagreements about the definition of overdiagnosis. Furthermore, in our paper, we do not propose any specific operationalization of overdiagnosis.

As the 2018 definition of Brodersen et al. (‘overdetection’ and ‘overdefinition’) [3] is very similar to that previously given by Rogers and Mintzker in 2016 (‘maldetection’ and ‘misclassification’) [4], both sets of authors might agree on its operationalization for measurement purposes. But what Rogers acknowledges (along with her co-authors here, Hofmann, Carter and Reid), and Gram et al. resist, is that this definition and its operationalization involve value commitments that make it inevitable that both the identification and mitigation of overdiagnosis must be worked out in processes that involve patients, professionals, and society at large.

Epidemiologists may continue to measure overdiagnosis in terms of expanding disease boundaries but surely they would like also to identify overdiagnosis within existing disease boundaries. They may continue to measure overdiagnosis in terms of the proportion of disease identified that does not progress, but determining how much non-progression is enough to make a diagnosis into an overdiagnosis is a value judgment. In our view, epidemiologists must be transparent with the public about this.

Disease definitions and boundaries inevitably entail considering both human values and biology; even mortality, perhaps the most basic outcome measure, is complicated by judgments about the relationship between length of life and quality of life; judgements involving values. Indeed, key elements of the authors’ definition require value judgements. Gram, Brodersen, and colleagues define ‘overdefinition’ as ‘the creation of new diagnoses by overmedicalising ordinary life experiences or expanding existing diagnoses by lowering thresholds or widening diagnostic criteria, without evidence of improved outcomes’. We respectfully note that what is an ‘ordinary life experience’ is a value judgment. This definition also leaves open the possibility that if there were evidence of improved outcomes (i.e. benefits), then lowering a threshold or widening diagnostic criteria would not be considered overdiagnosis. Thus, benefits are already implicit in Gram, Brodersen, and colleagues’ own definition, so if there is a problem with including benefits in a definition of overdiagnosis, this problem applies likewise to their definition.

In our article we acknowledge that defining, delimiting, and operationalizing overdiagnosis for measurement purposes is challenging. One reason for this is that the concept of overdiagnosis includes diverse phenomena with different types of uncertainty. These pose both conceptual and practical challenges for operationalization.

Our work in combating overdiagnosis would be easier if it were in fact a less complex concept than diagnosis, as Gram, Brodersen, and colleagues claim, but this seems implausible on the face of it. This complexity, however, is not a barrier to action to mitigate the harms of overdiagnosis. The now decades-old patient centred outcomes research movement shows that through high quality research and consensus-building processes, the experience and understanding of affected people can be captured and incorporated into outcomes measurement in a standardized way (see e.g. PCORI [5]). We strongly agree—as illustrated by the authors’ two examples of ADHD and prostate cancer—that taking the perspectives of affected persons seriously involves real challenges. But responding to overdiagnosis, in our view, requires a willingness to compare and constructively critique the perspectives of all stakeholders, including not only affected persons, but also researchers, clinicians, policymakers, companies, professionals, and institutions.

## Data Availability

No data referred to in the commentary.
